# Effect of Nitrogen Doping and Temperature on Mechanical Durability of Silicon Carbide Thin Films

**DOI:** 10.1038/s41598-018-28704-3

**Published:** 2018-07-11

**Authors:** Jan Tomastik, Radim Ctvrtlik, Tomas Ingr, Jan Manak, Ariana Opletalova

**Affiliations:** 1 Institute of Physics of the Academy of Sciences of the Czech Republic, Joint Laboratory of Optics of Palacky University and Institute of Physics AS CR, 17, listopadu 50a, 77207 Olomouc, Czech Republic; 2 Regional Centre of Advanced Technologies and Materials, Joint Laboratory of Optics of Palacky University and Institute of Physics of Academy of Sciences of the Czech Republic, Faculty of Science, Palacky University, 17, listopadu 12, 77146 Olomouc, Czech Republic; 3 Department of Experimental Physics, Faculty of Science, Palacky University, 17, listopadu 12, 77146 Olomouc, Czech Republic; 4 Department of Material Analysis, Institute of Physics CAS, Na Slovance 2, 18221 Prague, Czech Republic; 5 Regional Centre of Advanced Technologies and Materials, Faculty of Science, Palacky University Olomouc, Šlechtitelů 27, 78371 Olomouc, Czech Republic

## Abstract

Amorphous silicon carbide (a-SiC) films are promising solution for functional coatings intended for harsh environment due to their superior combination of physical and chemical properties and high temperature stability. However, the structural applications are limited by its brittleness. The possible solution may be an introduction of nitrogen atoms into the SiC structure. The effect of structure and composition on tribo-mechanical properties of magnetron-sputtered a-SiC_x_N_y_ thin films with various nitrogen content (0–40 at.%) and C/Si close to one deposited on silicon substrates were evaluated before and after exposure to high temperatures up to 1100 °C in air and vacuum. IR transmission spectroscopy revealed formation of multiple C-N bonds for the films with N content higher than 30 at.%. Improvement of the organization in the carbon phase with the increase of nitrogen content in the a-SiCN films was detected by Raman spectroscopy. Nanoindentation and scratch test point out on the beneficial effect of the nitrogen doping on the tribo-mechanical performance of a-SiC_x_N_y_ coatings, especially for the annealed coatings. The improved fracture resistance of the SiCN films stems from the formation of triple C≡N bonds for the as deposited films and also by suppression of SiC clusters crystallization by incorporation of nitrogen atoms for annealed films. This together with higher susceptibility to oxidation of a-SiCN films impart them higher scratch and wear resistance in comparison to SiC films before as well as after the thermal exposure. The best tribo-mechanical performance in term of high hardness and sufficient level of ductility were observed for the a-Si_0.32_C_0.32_N_0.36_ film. The enhanced performance is preserved after the thermal exposure in air (up to 1100 °C) and vacuum (up to 900 °C) atmosphere. Annealing in oxidizing atmosphere has a beneficial effect in terms of tribological properties. Harder films with lower nitrogen content suffer from higher brittleness. FIB-SEM identified film-confined cracking as the initial failure event in SiC, while it was through-interface cracking for SiCN at higher loads. This points out on the higher fracture resistance of the SiCN films where higher strains are necessary for crack formation

## Introduction

Operation conditions of new generation of advanced ceramic-based coatings and thin films often include harsh and high temperature oxidizing atmospheres or highly loaded mechanical contacts either during the fabrication process or the service life^1–3^. Therefore a high hardness ensuring the resistance to scratch initiation together with a sufficient amount of ductility preventing brittle large area failure^4–6^ combined with a high temperature oxidation resistance are of the highest importance for these coatings’ structural applications^7^. An intensive effort has been devoted to a research and development of ultra-durable coatings^8–13^. However, during the lifetime the elevated temperature exposure can lead to the chemical and structural changes of these coatings that result in change of mechanical and tribological properties. Hence the effect of thermal exposure on the coatings’ chemical and physical properties stability has to be carefully and reliably explored.

An exceptional rank in terms of structural ceramic belongs to SiC-based materials that are considered as a very promising candidate with an outstanding potential for diverse applications. This stems from strongly bonded three dimensional structure composed of light elements, resulting in high hardness^14^, superior tribological resistance^15^, chemical and mechanical stability even at elevated temperatures^16,17^ as well as high radiation resistance^18^. Moreover, appropriate doping strategies allow tailoring the optical, electrical and mechanical properties^19,20^. Especially the band gap engineering together with easy applicability of silicon-based technology make SiC an ideal material for electronic devices (MEMS, sensors transistors, etc.)^21,22^ used even in harsh environments^23–25^ as well as for applications in space optics and electronics^26–29^. The combination of exceptional high temperature mechanical durability with chemical and radiation resistance makes SiC a serious replacement material for zirconium-based alloys in case of cladding for nuclear fuel^30,31^ or a very promising material for the plasma facing components in nuclear fusion reactors^32–35^.

Despite the vast application potential of SiC its structural applications are limited by its brittleness, which has hindered its use in contrast to other more sturdy transition-metal carbides^36^ or nitride based ceramics^28^. It is especially due to the directional covalent bonds between Si and C atoms that do not allow dislocation based deformation^37,38^. Crystalline silicon carbide fractures transgranularly along the grain boundaries. Therefore, many efforts have been devoted to optimize the grain size^39^ shape^40^ or sintering additives^41–43^. There has been proposed several approaches to improve the toughness of ceramic materials both in bulk and thin films based on tailoring their composition and/or microstructure^44–47^. Toughness, in general, is defined as the ability of the material to absorb deformation energy before fracture and different methodologies have been developed based on (i) ductile phase incorporation, (ii) grain size refinement and reinforcement, (iii) structure grading^15,48^, (iv) multilayered concept^44,47^ taking advantage of crack deflection, crack tip blunting due to nanoplasticity and ductile interlayer ligament bridging at the interface between layers^49–53^ or (v) phase transition^54,55^. This can be accomplished either by the careful choice of technological process conditions^56,57^ or by changing the composition^36^ and/or by creation of specific structure^9^. The main process parameters of physical vapor deposition (PVD) of thin films includes electrical power, substrate temperature^56,57^, working gas^58^, deposition pressure, substrate bias, chamber design etc. In the alternative approach the chemical composition and in turn the mechanical properties of the Si-C-M system, where M stands for transition metal, can be modified depending on the C-M bond strength and the ratio of C/M atomic radii^59^. In this case the atoms are attracted by the mixture of covalent, ionic and metal bonds. Depending on the deposition conditions and actual composition various nanocomposite structures can be also formed. A typical example is nc-TiC_x_/a-SiC, where TiC_x_ nanograins are embedded in the amorphous a-SiC matrix. In general the Si often leads to reduction of grain size in nc-MC_x_/a-C system^36^. However the XRD amorphous structures were also reported for Cr-Si-C, Ta-Si-C, Mo-Si-C and W-Si-C systems^60–64^. Another promising nanostructure based on Si-C system may be represented by the Ti_3_SiC_2_ phase^65^ or Ti_4_SiC_3_^66^ MAX phases. These nanolayered hexagonal ceramics, following the general formula M_n+1_AX_n_ (where n = 1,2,3, M is an early transition metal, A is a group A element and X is carbon or nitrogen), combines the merits of metals and ceramic such as high hardness, good machinability together with enhanced ductility^66–70^.

The possible solution to overcome the high fragility of silicon carbide may be an introduction of nitrogen atoms into its amorphous structure. Especially the amorphous structure is important since in terms of enhanced toughness it is preferable over the crystalline one^46^. Besides, introduction of nitrogen into the Si-C system has been shown to be very promising for achievement of very high thermal stability above 1000 °C, where the transition metal nitrides suffer from extensive oxidation^71^. In fact the a-Si_x_C_z_N_y_ coatings were reported to possess different properties in comparison to the coatings based on mixture of Si_3_N_4_ and SiC and exhibit exceptional thermal stability even above 1500 °C that is considered as an ultimate temperature for Si_3_N_4_^72^. The potential of SiCN films has been explored extensively in last few decades^15,72–78^ and it was reported that the best mechanical properties exhibit films with composition along the SiC–Si_3_N_4_ line in the Si–C–N compositional triangle^74^ and that the highest thermal stability may be ascribed to the amorphous structure and Si-C bonds^73^. Besides, nitrogen doping has been already reported as a very convenient way to modify band gap of SiC_x_N_y_ (N-doped SiC) coatings^79^. In case of SiCN thin films and coatings, different approaches based on various types of physical vapor deposition (PVD)^19,20,73,80,81^ and chemical vapor deposition (CVD)^82–86^ techniques have been employed using different source materials (targets) and gasses (CH_4_, C_2_H_2_ or SiH_4_)^87,88^. Since these techniques may be operated under thermodynamically unstable conditions, it is possible to prepare materials with unique structures and compositions far from the thermodynamical equilibrium that are unattainable by standard bulk technologies^15,73,74,80,89–92^. Both PVD^90^ and CVD^87^ techniques in case of SiC_x_N_y_ thin films and coatings as well as polymer to ceramic transformation-based approaches for SiC_x_N_y_ bulk^93^ have succeeded in reaching exceptional thermal stability^72^. However high values of hardness approaching 20 GPa have been reported mostly for various PVD and CVD methods^73,80,87,88^. This clearly reflects the complexity in tailoring mechanical and chemical resistance and points out on the effect of conditions and peculiarities of the fabrication process on structure and composition of the SiC_x_N_y_ material.

Regardless of whether the primary application is based on optical, electrical or mechanical properties, also the protective ability, mechanical stability and durability of SiC or SiCN bulk/coatings are equally important. It should be noted that in case of thin films and coatings the strong adhesion to the underlying substrate is also of the highest importance as evidenced above. There have been introduced tens of various tests for evaluation of adhesion/cohesion strength^94,95^, but in case of layered systems the scratch test has been established as the most reliable and recommended one^96,97^. This is especially due to its simple principle, high reproducibility and ability to mimic the service load conditions. Despite the vast application potential and already proved applicability of SiC and/or SiC_x_N_y_ there is a lack of systematic data on the adhesion strength of these coatings and only few studies can be cited^33,87,88,98–100^. This is further underlined by the fact that the adhesion failure is often the primary failure mechanism of coatings^101^. The wear behavior of amorphous Si/C/N:H synthesized by RF plasma enhanced chemical vapor deposition (RF-PECVD) was studied in sliding tests under oscillating motion and brittle nature combined with rather week adhesion were reported^87,88^. The adhesion and failure mechanism of RF magnetron sputtered SiC thin films on steel^33^ and nanocrystalline Si-C-N films on Si were also reported^98,99^ and compared to a-CN_x_ in latter case.

In previous studies we deposited a-SiC_x_N_y_ films with various N content ranging from 0 to 40 at.% and thoroughly analyzed their structural and compositional stability after air and vacuum annealing^80,90^ as well as the high temperature mechanical properties^91^. This paper expands the scope of earlier works by focusing on the adhesion and tribological performance of these amorphous SiC_x_N_y_ coatings before and after high temperature annealing at 700, 900 and 1100 °C both in air and vacuum and relate them to structure, composition and mechanical properties. The standard ramped scratch test and repetitive scratch test (multi-pass wear test) were employed to explore both the effect of N content and the annealing temperature. In addition, the methodology of nano/micro scratch test evaluation is reported and the extraordinary performance of acoustic emission-based approach is demonstrated.

## Experimental

### Thin films

Amorphous SiC_x_N_y_ films were deposited using reactive direct current (DC) magnetron sputtering on Si(111) substrates using Leybold-Heraeus Z 550 M sputtering apparatus. Hot-pressed SiC target was sputtered in argon and nitrogen-argon mixture with different N_2_/Ar flow ratios (0, 0.04, 0.08, 0.16, 0.32, and 0.48) resulting in six coatings with different chemical composition (see Table 1, for more information see^80^). The chamber base pressure was set to 5 × 10^−4^ Pa, while the deposition working pressure varied between 0.5–0.6 Pa. The discharge power was kept 320 W and the N_2_/Ar flow ratio was governed by varying the N_2_ flow rate (0, 1, 2, 4, 8, and 12 sccm) while maintaining stable Ar flow rate at 25 sccm. Distance between substrate and target was 50 mm. A bias voltage of about −60 V was applied to substrate holder using a 13.56 MHz power source in order to deposit films with dense structure based on previous experiments^56,80,89^. Multiple samples were always deposited during one deposition run at a specific N_2_/Ar ratio.

The as deposited samples were then annealed in a furnace with air atmosphere at temperatures of 700, 900 and 1100 °C for 30 minutes; each sample was annealed only at one temperature. Heating rate was set around 20 °C/min, while cooling occurred spontaneously. In addition, a set of samples was also annealed at 900 °C in the vacuum to avoid the surface oxidation and separate the effect of temperature on structure, mechanical and tribological properties. This was motivated by the fact that thermal resistance actually includes several aspects like chemical or structural stability, oxidation resistance and endurance against thermal decomposition. Moreover, there is a significant influence of the used atmosphere. For instance a bulk SiN ceramic decomposes at 1400 °C in vacuum, while the ultimate temperature increases to 1775 °C in 0.1 MPa N_2_ atmosphere^93^.

### Analytical methods

Structure of all as deposited and annealed samples was analyzed using micro-Raman spectroscopy and IR spectroscopy. The former was use in backscattering configuration on Renishaw Ramascope, Model 1000 with the exciting laser beam at 514.5 nm (6 × 10^4^ W/cm^2^). Transmission IR spectra were obtained via Fourier-transform spectrometer Bruker IFS 113 v. Composition of the as deposited films was measured using electron probe X-ray microanalysis (EPMA) with JEOL JXA-733 apparatus equipped with the microanalyzer Kevex Delta Class V. Energy dispersive spectral analysis of scanning electron microscope (SEM-EDS) was used for observation of residual grooves after scratch tests in order to study the oxidation layer. Thickness of the films was measured using a mechanical profilometer and a laser scanning confocal microscope Olympus OLS LEXT 3100 that was also used for observation of surface topography and evaluation after tribological tests. Surface roughness was measured via AFM in a contact mode with the CSG cantilever using NTEGRA Spectra instrument (MDT).

Mechanical and tribological properties were tested on MicroMaterials NanoTest NTX apparatus. Indentation loads of 10 and 50 mN applied to the diamond Berkovich indenter were used to obtain hardness and reduced elastic modulus in different depths. Adhesion/cohesion properties of film/substrate systems were explored using two scratch test variants. First, the ramped scratch test with linearly increasing load up to 500 mN aimed to identify the critical loads (*L*_c_) for cohesive and/or adhesion failures. The test was performed as a three-scan procedure – first topography scan is followed by on-load scratch over the same track, after which the surface is scanned by the final topographic pass. Second, the repetitive wear test based on repetition of 10 subcritical on-load scratches (labeled as S) at constant load of 300 mN was used to evaluate the extent of elastic and plastic deformation as well as wear rate of the material. During the test each two on-load passes were followed by the topography pass (labeled as T). The test is often evaluated on the basis of the number of on-load passes without the traces of failure and can be therefore considered as a low-cycle wear test^102^. At least four ramped tests and two wear tests were carried out in all samples.

The ramped scratch tests were performed with the 5 µm nominal radius sphere, while the repetitive scratch test with blunter 10 µm nominal radius sphere. The sharper 5 µm indenter had to be used to initiate the film failure since 10 µm tip did not provide sufficiently high strains for films fatal failure during the ramped test. On the other hand the blunter tip is more appropriate for wear testing as explained later.

Evaluation of the ramped and repetitive scratch tests was performed using a combination of the depth change record corrected for frame compliance, microscopic observation of the residual wear tracks and the acoustic emissions record (AE). The acoustic emission signal was sampled at 2 MHz rate simultaneously during the whole test using a DAKEL Zedo AE system. The very high sensitivity of the AE system allowed detection of even the weak subsurface cracking in the film-substrate system that is undetectable by the traditional methods^103^.

To understand the response of SiC and SiCN thin films to the scratch loading the residual scratch grooves were cross-sectioned using the Focused Ion Beam milling with the FEI Quanta 3D Dual-Beam SEM/FIB system utilizing Ga^+^ ion source. Various perpendicular and longitudinal cross sections were prepared to reveal the dynamics of the material response.

### Data availability statement

All measurement data introduced in tables and graphs are available from corresponding author on reasonable request.

## Results and Discussion

### Structure and composition

The composition of the sputtered films was governed by the N_2_/Ar flow rate ratio during deposition resulting in the films with different N content up to 40 at.% and C/Si close to one. The thickness of the as deposited films increases from 2.2 µm for the films deposited in pure Ar to 2.7 µm for the one deposited at the highest N_2_/Ar ratio of 0.48. The changes in film thickness reflect the increase in deposition rate along with the increase of N content, as can be seen in Table 1, where also the sample labeling is introduced for the narrative simplicity. The surface of the films was mirror-like with arithmetic roughness Ra around 1 nm as measured using AFM.

**Table 1:** Films’ parameters after deposition.

Sample	Deposition gases ratio	Film thickness	Film chemical composition		
			Si	C	N
	N_2_/Ar	(μm)	(at.%)	(at.%)	(at.%)
00SiC	0	2.2	46	54	0
04SiCN	0.04	2.3	41	45	14
08SiCN	0.08	2.4	37	43	20
16SiCN	0.16	2.6	36	37	27
32SiCN	0.32	2.7	32	32	36
48SiCN	0.48	2.7	30	30	40

Air annealing resulted in surface oxidation and in turn to increase of oxygen content in the films and also to color change. The films became darker with increasing annealing temperature and N_2_/Ar ratio. The susceptibility to oxidation, represented by the rise of the oxygen content, grows with annealing temperature, when it is only moderate at 700 °C. Surface oxidation always proceeded more extensively at higher temperatures (above 1000 °C) and especially for the films with originally higher N content. Silicon content remains nearly the same, while C and N atoms are released at the expense of oxygen as reported by Kulikovsky and Ctvrtlik^80,90^.

**Figure 1:**
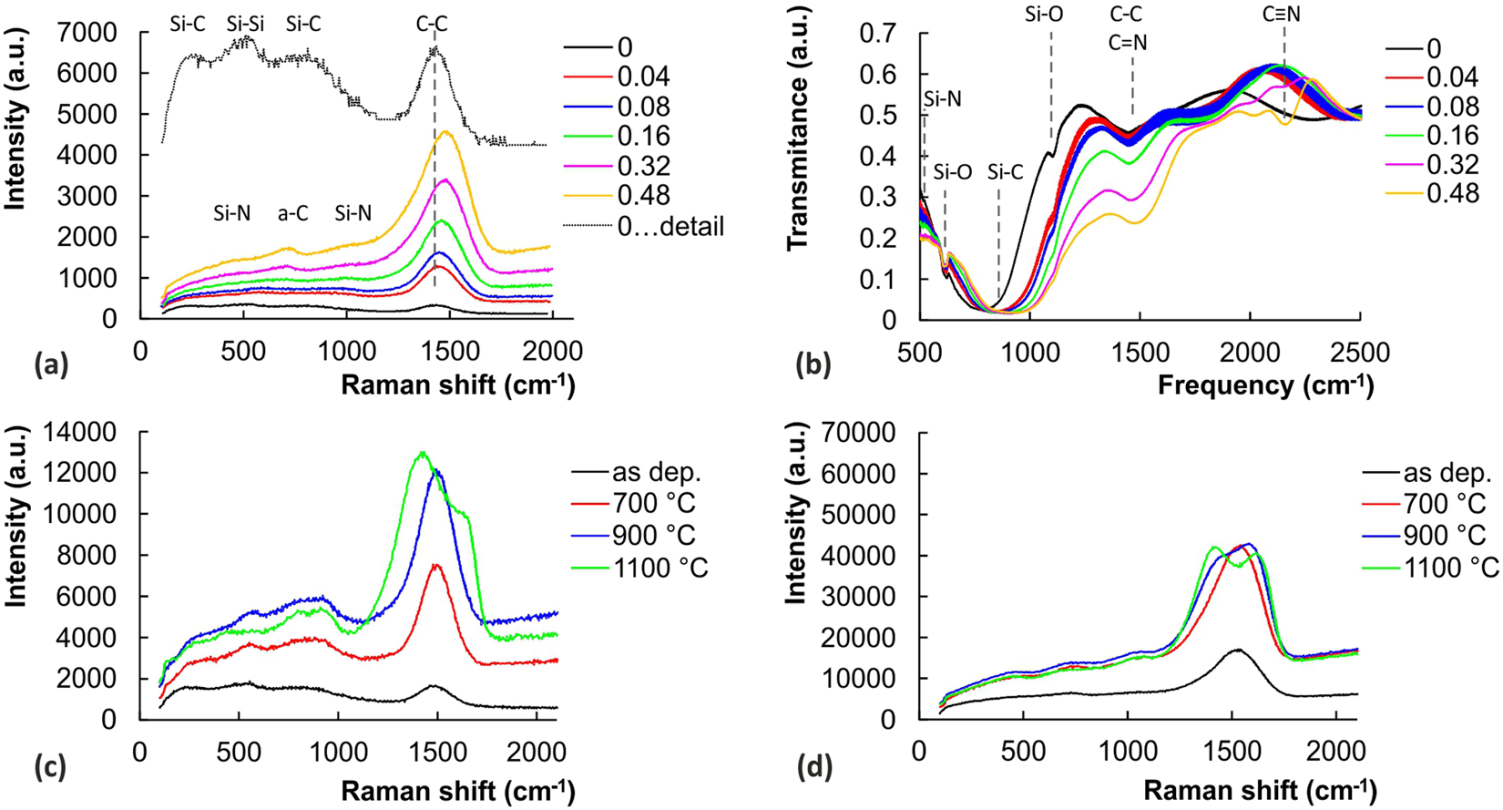
IR transmission and Raman spectra for (**a**,**b**) all the as deposited a-SiCN films and (**c**,**d**) SiC and Si_0.32_C_0.32_N_0.36_ film deposited at the N_2_/Ar = 0.32 with originally 36 at.% of N. Note the different scale in (**c**,**d**), detail of the SiC films is presented with extended y-axis is presented in (**a**)^80^.

Micro-Raman spectroscopy (Fig. 1) showed that in the 00SiC film the Si-dominant and C-dominant regions coexist with SiC clusters as typical for sputtered SiC films^56^. Introduction of nitrogen into the film system leads to the creation of C-N and Si-N bonds. Mainly the later are favorable due the higher difference in elements’ electronegativities. The overall increase of scattering intensity can be also seen. This effect is especially strong for carbon related bands that also up-shift and can be attributed to the increase of amount of C-N and C=N bonds^80^. The Si-C bands are suppressed reflecting lower amount of Si-C bonds at the expense of Si-N bonds as further supported by the IR spectra. In general, the higher intensity of the carbon related bands in Raman spectra is related to the much higher polarizability of C-C bond in comparison to Si-C, Si-Si and eventually to Si-N bonds^80^.

Annealing of the films led to the short range ordering in clusters; nevertheless their amorphous structure was preserved, except the 00SiC film where traces of crystalline SiC phase were observed after annealing at 1100 °C, see apparent splitting of the Si-C band in to components at 780 and 900 cm^−1 ^^80,104^. This indicates the formation of nanocomposite structure composed of SiC nanocrystals and a-C phase, similarly to^56^. Formation of such a nanocrystalline microstructure is probably responsible for the increased hardness and reduced modulus of these films as shown later. Pronounced ordering was observed especially in carbon clusters where graphite-like structure was developed. This process started already at 900 °C for the films with higher nitrogen content, compare Fig. 1c,d. Films’ thickness remained practically stable with the change no higher than 4%. More information about detailed analysis of the films’ structure and can be found in our previous work^80^. Annealing state will be reflected in the following text into the samples’ labeling.

The transmission IR spectra for all the as deposited films with labeled characteristic band are shown in Fig. 1b. Spectrum for SiC film features strong band around 780 cm^−1^ and 1450 cm^−1^ characteristic for Si-C and C-C bonds, respectively. Besides, two weak bands corresponding to the Si-O vibration from a native silicon oxide on the Si substrate can be identified around 610 cm^−1^ and 1100 cm^−1^. Introduction of N atoms is accompanied with the broadening and upshift of the Si-C band to 850–910 cm^−1^ due to the formation of Si-N bonds^105^ as N replaces C. Existence of this broad band imply that the compact amorphous Si-C-N structure is formed. Strong decrease in transmittance around 500 cm^−1^ also evidences formation of Si-N bods, especially for films with higher N content. Similarly, the drop in transmission for the band at 1450 and its upshift is apparent and may be connected to the increase of C=N vibrations. This effect corresponds to the strong increase in scattering intensity of main carbon band in Raman spectra. On the other hand, in contrast to Raman spectra, the triple C≡N bond is observed for the SiCN films with the highest N content above 36 at.% as evidenced by the bands around 2175 cm^−1^ ^106^. It should be noted that especially the double and triple bonds have a considerable effect on mechanical and tribological performance of the SiCN films. Especially the triple C≡N bond is crucial as it prevents from crosslinking of the C atoms and formation of strong 3D structure.

### Mechanical properties

Thorough analysis of mechanical properties can be found in our previous publication^80^, here only the main findings related to the tribology and necessary for complex understanding of films’ durability and performance are summarized. Hardness (*H*) and reduced elastic modulus (*E*_r_) values of the as deposited films decline with the increasing content of nitrogen in the films, specifically from *H* = 23 GPa and *E*_r_ = 293 GPa for the pure 00SiC film to *H* = 18 GPa and *E*_r_ = 242 GPa for 48SiCN film with the highest nitrogen content. Both values are given from the indentation at the lowest load of 10 mN.

**Figure 2:**
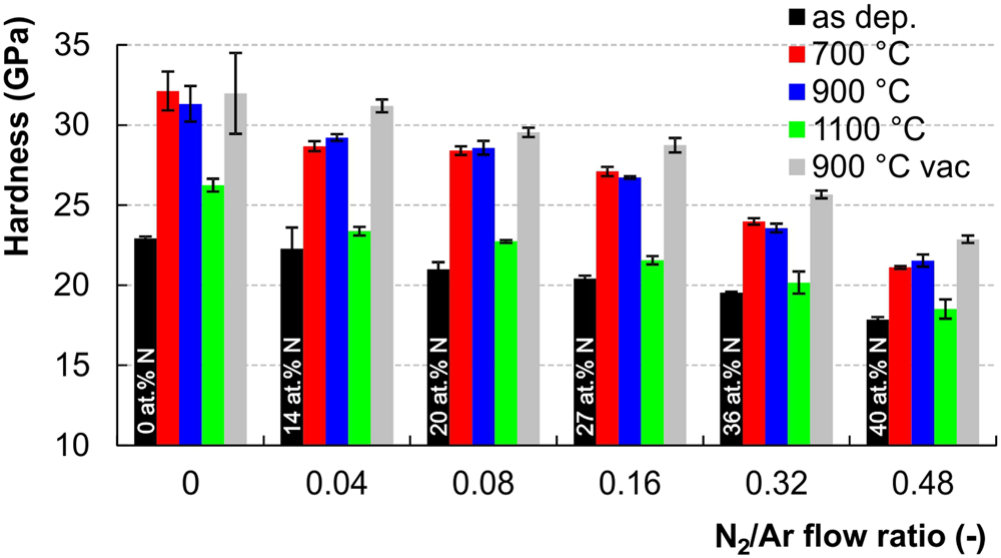
Hardness values for complete set of a-SiC_x_N_y_ samples from 50 mN indentations.

In order to explore the effect of annealing on mechanical properties the hardness values measured at load of 50 mN are compared in Fig. 2. The increased indentation load was used to minimize the effect of soft SiO_x_ layer formed during air annealing. This allowed obtaining more relevant hardness values reflecting the short range ordering of the SiC_x_N_y_ films as more information comes from the unoxidized volume. It should be noted that for the as deposited, 900 °C vacuum and 700 °C air annealed films the hardness values for 10 and 50 mN are almost identical.

Air annealing at 700 °C leads to hardness increase in all samples. This effect was more pronounced for the films with initially lower nitrogen content (see Fig. 2). Comparing to the as deposited films the hardness increase of 40%, 29%, 35%, 33%, 23% and 18% can be observed for the films ranging from 00SiC700 to 48SiCN700. After air annealing at 900 °C hardness of the inner layers remains almost unchanged, as can be seen from Fig. 2. Increase of the annealing temperature up to 1100 °C on the other hand leads to small decrease in hardness values due to more pronounce effect of thicker oxidized SiO_x_ layer. It should be noted that the top surface hardness drops even to the values typical for fused quartz (SiO_2_) around 9 GPa, as evidenced by the additional experiments at very low load of 0.5 mN.

The pure effect of temperature on mechanical properties was investigated by vacuum annealing at 900 °C. Without the surface oxidation-related softening only the short range ordering took place and cause a dramatic rise of hardness in comparison to as deposited films. The highest hardness increase was observed for the pure 00SiC900vac films and reaches 45%, while the lowest of 29% for the 48SiCN900vac film deposited at the highest N_2_/Ar ratio. At the same time practically no difference in hardness values was observed between 10 mN and 50 mN indentations.

All the above-mentioned findings reflect a different hardening potential of ternary a-SiC_x_N_y_ compounds induced by thermal treatment in terms of N content. Especially ordering in SiC clusters and improvement of Si-C bonds is responsible for hardness and modulus growth on one hand, while it is formation of multiple C=N and C≡N bonds terminating the 3D network decreasing the potential of structural strengthening on the other hand^107,108^. It should be noted that the plasticity index, defined as the ratio of the plastic indentation work to the total indentation work^108^, slightly increases with the increase of N content for the as deposited and remains preserved even after annealing.

Elastic modulus of the as deposited SiCN films decreases with the growth of N content. Taking into account the shallowest indents performed at 10 mN, the values declines from 293 GPa for 00SiC to 242 GPa for 48SiCN with approx. 40 at.% of N. Air as well as vacuum annealing at 700 °C and 900 °C promotes the short range ordering and leads to increase in elastic modulus for the films deposited at N_2_/Ar up to 0.16. However, no increase was observed for the films deposited at N_2_/Ar higher than 0.16. After annealing at 1100 °C the modulus values dramatically dropped for all the SiCN films even below the value for as deposited films. On the contrary, the elastic modulus of the 00SiC film saturates^80,90^.

#### Ramped scratch tests

Ramped scratch tests were performed under the same conditions in four repetitions on all samples so the results can be directly compared each other. The position of the von Mises stress maximum for the maximal load of 500 mN with the use of 5 µm tip radius was calculated to be around 1400 nm; that is well within the film thickness, ensuring the films’ properties were primarily tested. Two types of critical loads representing the onsets of specific failure modes in the film-substrate system were identified using the combination of three independent evaluation methods. The first critical load *L*_C1_, exclusively determined via AE method, marks the initial cracking in the film-substrate system. The second critical load *L*_C2_ indicates the full delamination of the film leading to uncovering of the substrate and was determined using the combination of depth change record and microscopic observation of the residual groove.

**Figure 3:**
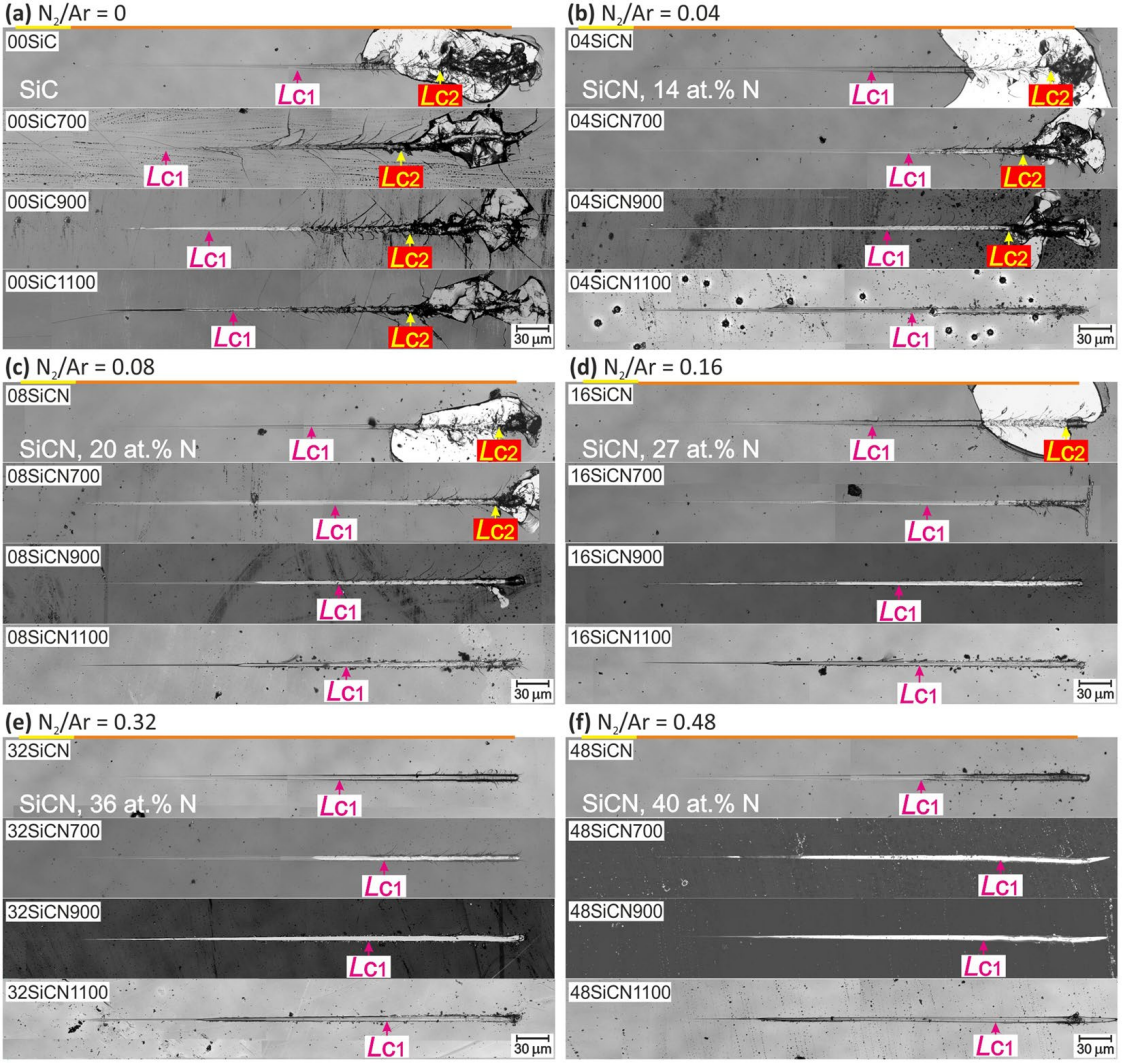
Typical residual scratch grooves after progressive load scratch test up to 500 mN on each sample from 00SiC to 48SiCN films before and after annealing at 700, 900 and 1100 °C in air.

Two independent effects of (i) annealing temperature and (ii) N content (0–40 at.%) on the tribological response of SiC and SiC_x_N_y_ are explored. Therefore the results are discussed from both perspectives. The representative residual grooves for each sample are shown in Fig. 3 where the as deposited and 700, 900 and 1100 °C air annealed films are grouped based on N_2_/Ar flow rate ratio. In general, the morphology of the individual residual scratch grooves was almost identical that reflects a good homogeneity of the films and also a high repeatability of the test itself.

Taking the annealing temperature as a parameter, one can compare different evolution of tribological response of films with given compositions controlled by N_2_/Ar ratio during deposition. The significant films’ delamination extending beyond the residual groove can be seen for all 00SiC films (N_2_/Ar = 0), see Fig. 3a. As deposited 00SiC sample exhibits gross spallation in backward direction from the *L*_C2_ point (further explained in section *Dynamics of the failures in the film-substrate systems*). The arc tensile (inside the scratch groove) and angular (spreading out of the scratch groove) cracks formed behind the moving indenter are the main crack type between both critical loads. In general, the angular cracks are considered to be formed due to the combination of parallel (behind the moving indenter) and perpendicular (around the moving indenter) tensile stresses^109^. The angle of angular cracks is governed by the superimposition of these two stress fields. Similar angular cracks were reported for instance for WC-CoCr and DLC films or Ti-6Al-4V^109–112^. Annealing at 700 °C in air led to an apparent change in the response to scratching stress. Shortly after *L*_C1_ a tensile and later angular cracks spreading out of the residual scratch groove became dominant crack type. Delamination of this 00SiC700 film was more constrained. Further increase of annealing temperature changes the length and frequency of angular cracks. This can be attributed to partial relaxation of internal compressive stress and/or partial crystallization in SiC clusters and formation of nanocomposite microstructure during annealing similarly to^56^.

Introduction of N into the films promotes gradual reduction of fragility of the SiCN films, especially for higher N content. The role of N atoms is twofold – they hinder organization in SiC clusters and facilitate graphitization in carbon clusters^80,90^. The as deposited films 04SiCN, 08SiCN and 16SiCN with N content of 14, 20 and 27 at.% reported gross backward spallation and crack patterns in the revealed substrate similarly to 00SiC, see Fig. 3. Angular cracks are also clearly visible, but their length and intensity are significantly reduced with the annealing temperature. The level of scratch cracking resistance grows with the increase of N content and reflects the increased films’ toughness. It can be clearly seen that the catastrophic film failures are not observed for higher annealing temperatures for films with higher N content. For example the catastrophic film failure is observed for 04SiCN film after air annealing at both 700 and 900 °C, while in case of 08SiCN film only after annealing at 700 °C, none for the annealed cases of 16SiCN film. The improved scratch resistance of the annealed films stems from the formation of softer SiO_x_ surface layer that acts as a tribolayer and changes the stress distribution considerably^113–115^. The growth of the oxide layer is more pronounced for higher temperature annealing. The thickness of the SiO_x_ layer of (70 ± 20) nm was measured independently using the confocal microscopy and SEM.

**Figure 4:**
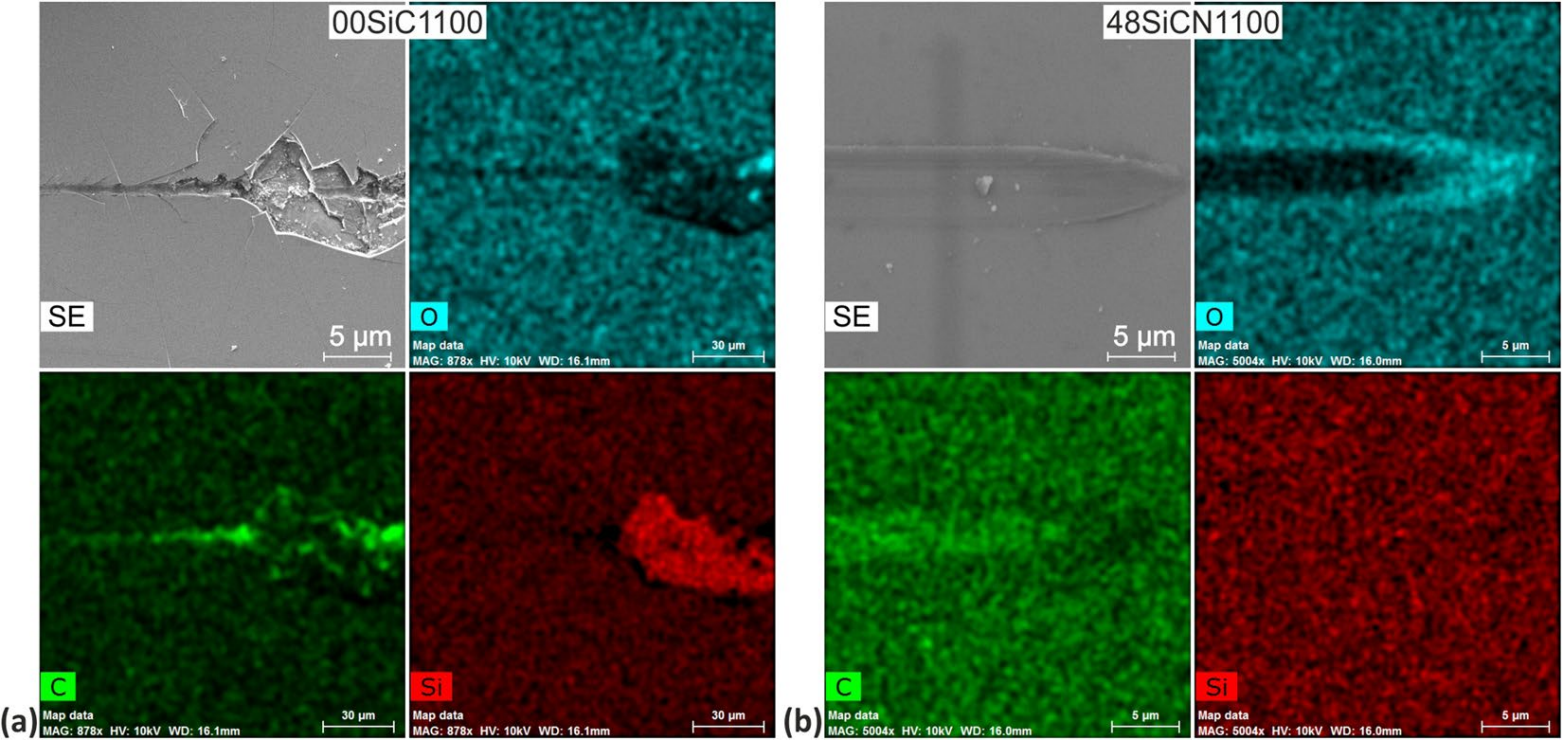
SEM-EDS map of chemical elements from the final parts of residual scratches. (**a**) 00SiC1100 without nitrogen and (**b**) 48SiCN1100 with maximum nitrogen content after 1100 °C annealing. Oxygen, carbon and silicon maps are presented.

Significant change in comparison to previous group can be seen for samples 32SiCN and 48SiCN as scratch tests did not cause gross spallation even in as deposited films (see Fig. 3e,f). This clearly reflects the effect of multiple C-N bonds and formation of amorphous Si-C-N, see Fig. 1. Besides, the graphitization together with lower level of internal stress also contribute. Both as deposited films exhibited only minor tensile cracks inside the residual groove and faint angular cracks. After annealing at 700 °C the angular cracks expanding well beyond the edges of the residual groove were clearly detected only for the 32SiCN700. The onset of the angular cracking corresponds to the first failure in the film-substrate system (i.e. *L*_C1_) identified by an acoustic emission record in this case. In contrast, only arc tensile cracks were observed for 48SiCN700. Air annealing at higher temperatures of 900 and 1100 °C further enhanced the cracking resistance. Plastic deformation and wiping of the oxide layer can be seen without observable tensile cracks in the residual scratches as evidence in Fig. 4. The thickness of the oxide layer is larger for the samples deposited at higher N_2_/Ar that is also reflected in their improved scratch resistance.

**Figure 5:**
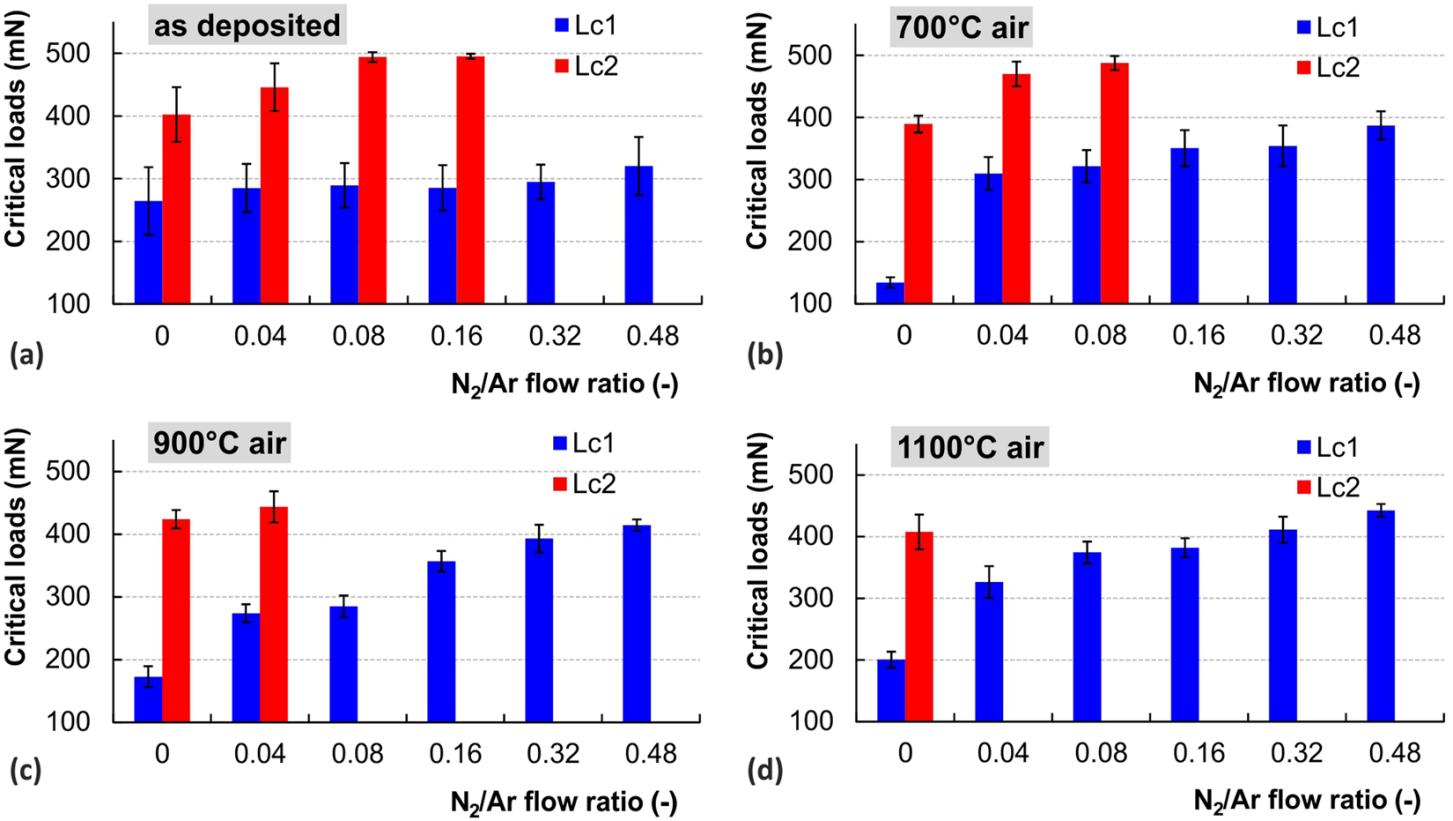
Summarizing critical loads for all samples from the view point of composition change in the given annealing parameters. N_2_/Ar flow ratio corresponds to the films’ chemical composition.

To quantify the ramped scratch test the critical loads were evaluated based on the depth change record, microscopic observation and the acoustic emissions. Figure 5a summarizes the critical loads, calculated as the average for at least four scratches, of all the as deposited films with variable nitrogen content. The trend of growing resistance of the films with higher nitrogen content is clearly noticeable as the values of both critical loads increase. The critical load *L*_C2_, representing the gross spallation, was observed only for the films with N content up to 27 at.% (00SiC-16SiCN). Films with higher nitrogen content did not exhibit such catastrophic damage that reflects their higher durability. On the other hand cracking in film-substrate system detected by the acoustic emission detection (labeled as *L*_C1_) was presented for all the films.

All the SiC_x_N_y_ films increased their durability after 700 °C annealing as manifested by the rise of both critical loads *L*_C1_ and *L*_C2_, see Fig. 5b. On the other hand, the pure SiC film shows a significant deterioration of its scratch resistance after annealing as reflected by the drop of *L*_C1_.

Further increase of annealing temperature to 900 °C led to apparent increase of *L*_C1_ for the SiC_x_N_y_ films with higher N content, see Fig. 5c. The onset of gross spallation, represented by *L*_C2_, was observed only for 00SiC900 and 04SiCN900 samples with the lowest N contents. Annealing at the highest temperature of 1100 °C in air is accompanied by the pronounced oxidation that further increases the *L*_C1_ values for all samples. This can be related to the formation of SiO_x_ tribolayer that forms more easily for films with higher N content due to their higher susceptibility to oxidation^80^. It is worth noted that the Raman spectra revealed crystallization in SiC clusters after annealing, see Fig. 1. It is believed that these small crystallites with nanometer size^56^ play a crucial role during the stress redistribution process under the moving diamond indenter and act as a stress concentrators^116^. The cracks are then initiated at the SiC crystallites and Si-N-C amorphous matrix interface^19,80^. No crystallization in SiC clusters was observed for SiCN samples, which may explain their better scratch durability.

#### Vacuum annealing

**Figure 6:**
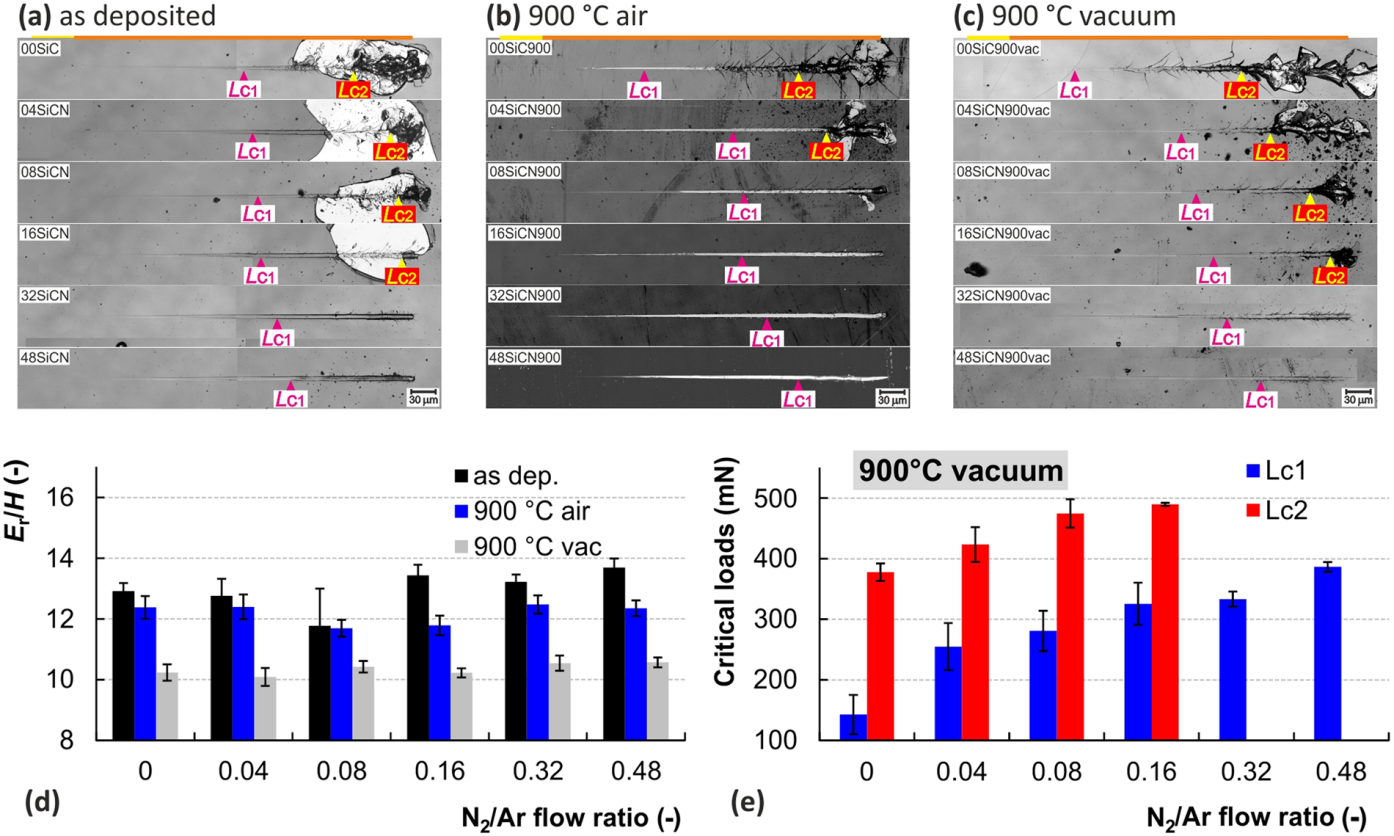
Results of vacuum annealed samples in comparison to other samples. Residual grooves for (**a**) as deposited films, (**b**) 900 °C air annealed films and (**c**) 900 °C vacuum annealed films show apparent differences in their failure modes. Graph (**d**) represents the *H*/*E*_r_ ratio and (**e**) shows the critical load of vacuum annealed samples, that complements the data from the Fig. 5.

In order to study the impact of thermally activated structural changes on the durability of a-SiC_x_N_y_ without the influence of SiO_x_ top surface layer the films were annealed in vacuum at 900 °C. Comparison of critical loads (see Fig. 6e) points out on slight decrease of *L*_C1_ and *L*_C2_ values relative to all 900 °C air annealed films (see Fig. 5c). On the other hand, in comparison to the as deposited films (see Fig. 5a) one can see decrease of *L*_C1_ only for the films with lower nitrogen content (00SiC, 04SiCN, 08SiCN), see Figs 5a and 6e. Angular cracks spreading out of the residual groove can be observed on the surface of all vacuum annealed samples closely after *L*_C1_ points. Their length is decreasing with increasing nitrogen content, which copied the trend of the as deposited samples, see Fig. 6a,b. In contrast to as deposited films, the gross spallation was not observed for vacuum annealed films similarly to air annealed samples.

The 900 °C vacuum annealed samples generally show lower durability in comparison to their air annealed counterparts, as evidenced by the earlier onset of formation and propagation of angular cracks, see Fig. 6. Wedge spallation is clearly recognized for the films 00SiC900vac, 04SiCN900vac, 08SiCN900vac and 16SiCN900vac deposited at values of N_2_/Ar = 0 to 0.16. The extent of this type of failure declines with the increase of N content in the films and its onset shifts gradually to the terminal parts of the scratch groove. According to Evans, the compressive shear crack formation followed by the interfacial detachment are two distinct processes occurring during wedge spallation^117^. The *L*_C1_ critical loads are lower due to their higher brittleness, despite the increased hardness of all vacuum annealed films in comparison to their air annealed counterparts (see Fig. 2). This may be attributed to the different stress redistribution and accommodation mechanics coming from the structure short range ordering. It also clearly illustrates the beneficial role of the SiO_x_ oxide tribolayer, formed during air annealing, and its positive effect on enhanced scratch resistance of the thin film.

The increased susceptibility to angular cracking of the vacuum annealed films can be explained by the decrease of *E*_r_/*H* ratio in comparison to the as deposited films or air annealed films at 900 °C. It is well known that the fracture toughness of brittle materials, related to the “critical stress intensity” for crack propagation^118^, is empirically linked to the hardness to elastic modulus ratio^119,120^. Various models for indentation fracture toughness have been proposed and *E*_r_/*H* appears in the majority of them^121,122^.

Taking into account the *E*_r_/*H* values calculated for the shallowest 10 mN indents, one can clearly distinguish between the vacuum and air annealed samples. The lowest cracking resistance of the vacuum annealed films apparent from Fig. 6 may be directly related to the lowest values for *E*_r_/*H* and hence the easier crack propagation. Although the oxidation top layer affects the hardness and modulus values, the above considerations can provide a useful indication of fracture susceptibility during single asperity scratch contact.

#### The dynamics of the failures in the film-substrate systems

The strength of the combined method based on the mutual evaluation and comparison of the visual observation, depth change record and acoustic emissions record can be clearly illustrated for the as deposited 00SiC thin film. The mostly used microscopic observation of the residual groove is a well adopted approach capable of distinguishing between different types of failure and hence provides a quantitative evaluation. Nevertheless the sole visual observation can be insufficient and can lead to inadequate results. For example it cannot take in to account the subsurface failures in case of non-transparent films, as discussed previously for Fig. 3d–f.

**Figure 7:**
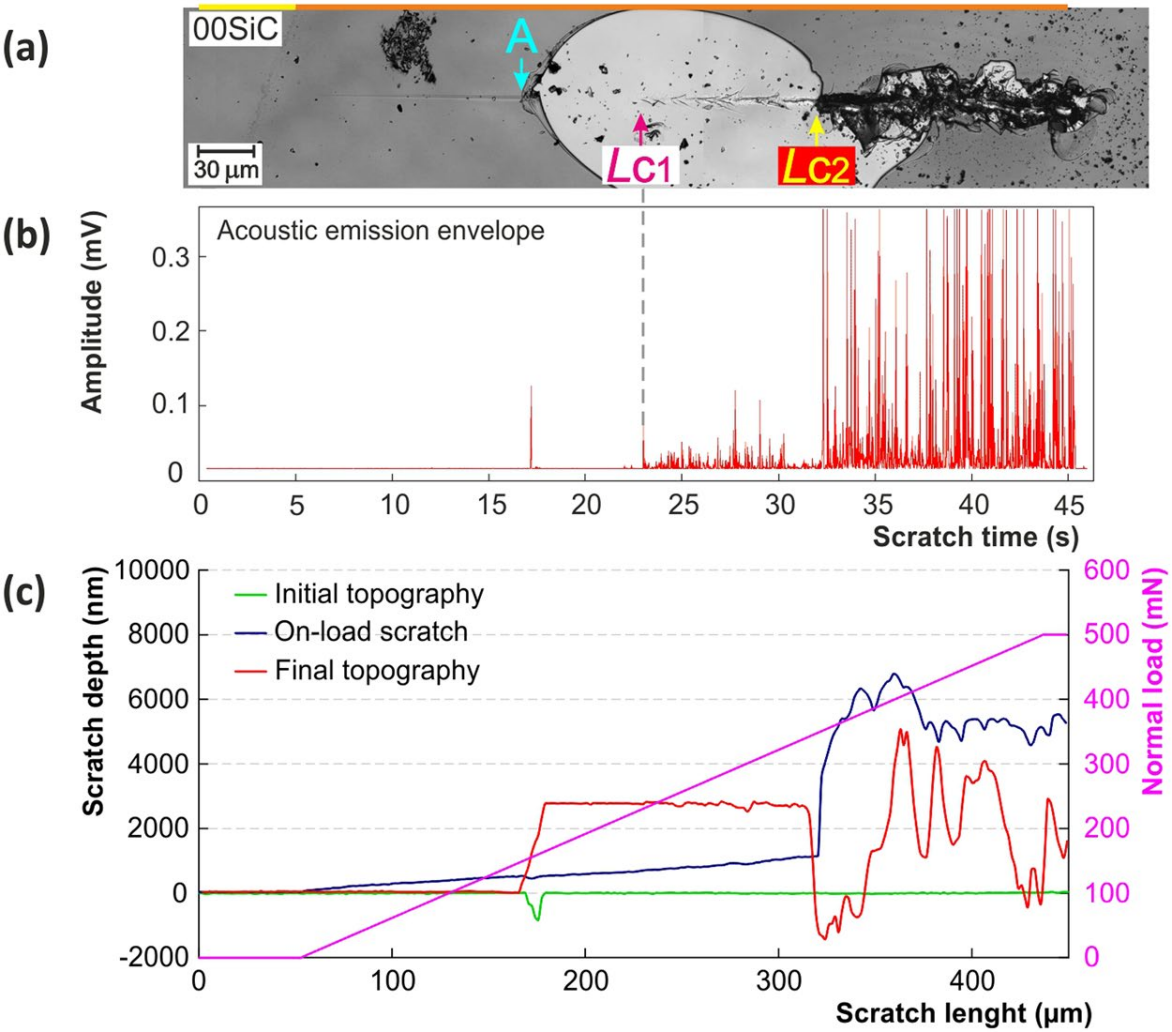
Advanced analysis of two ramped scratch tests on 00SiC sample with (**a**) the microscopic observation of residual groove, (**b**) the acoustic emissions record and (**c**) depth change record.

The residual scratch groove for the as deposited SiC film is shown in Fig. 7. Only the microscopic evaluation of this particular scratch would lead to the incorrect marking of the initial film failure to the point “A”. The actual dynamics of the film/substrate system failure can be elucidated with the help of the depth change record (see Fig. 7c), where the combination of the on-load curve (blue) and the final topography curve (red) indicates gross spallation originating from *L*_C2_ point, which expanded backward to the point “A”.

Besides, the exclusive information about the gradual film-substrate system damage can be obtained from the analysis of the AE signal recorded during the whole test, see Fig. 7c. A single peak can be seen around 17th second from the beginning of the test that represents cracking event originated at the surface defect that was actually confirmed in the initial topography pass (see yellow curve in Fig. 7c). The catastrophic film failure first occurs approx. 33 seconds from the beginning of the test as the backward gross spallation (marked as *L*_C2_). From this moment on, the pronounced cleaving starts in both the film and the substrate which is accompanied by abrupt increase of the AE signal amplitude. The advanced analysis in both the time and frequency domain of the AE hits, representing the individual cracking events, allows to clearly distinguishing between the film and the substrate cracking. The AE signal between 23rd and 33th second may be related to the substrate cracking as is confirmed in Fig. 7b, where the large substrate area is exposed due to the abovementioned large backward spallation. The onset of the visually detected substrate cracking (marked as *L*_C1_) fully agrees with the onset of the acoustic emission signal, compare in Fig. 7a and in Fig. 7b. It should be noted that on-load depth change record (blue curve in Fig. 7c) did not reveal any traces of cracking around *L*_C1_. Similar behavior, where film-substrate interface cracking is accompanied by AE signal, was observed for all the samples as supported later by FIB.

**Figure 8:**
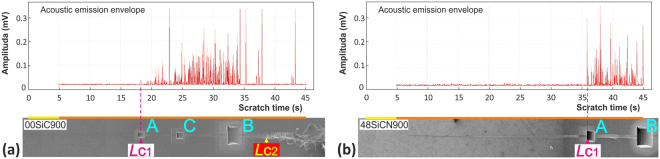
Overview of residual scratches and AE records of (**a**) 00SiC900 and (**b**) 48SiCN900 samples with marked areas of FIB cross-sections selected with regard to the position of critical loads that are also labeled.

The FIB milling was used to explore the response and type of cracking for pure SiC films and SiCN film deposited at N_2_/Ar = 0.48 and annealed at 900 °C in air. The perpendicular cross-sections were milled in two distinctive areas based on AE signal analysis, see Fig. 8. First, the cross-sections, labeled as “A”, were milled in the area corresponding to *L*_C1_ representing the onset of AE signal. Second, the cross-sections, labeled as “B”, were prepared in the areas with large cracking. In case of SiC film this was slightly before the *L*_C2_ representing catastrophic damage of the film. Final part of the scratch groove was chosen for the 48SiCN900 film since only here the faint cracks were observed. Since different crack types were identified for the 00SiC900 film, the scratch groove was sectioned in the intermediate area labeled as “C” with more pronounced cracking, see Fig. 8

**Figure 9:**
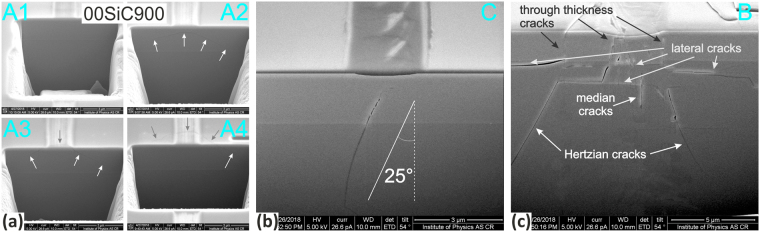
FIB cross sections in the 00SiC900 sample corresponding to the (**a**) the initial *L*_C1_ cracking, (**b**) developed cracks in the latter part and (**c**) the distorted area just before the film delamination (the *L*_C2_).

Figure 9a shows details of the brittle cohesion/arc crack induced by the tensile stress behind the moving indenter. The crack extends into the film in the backward direction and terminates at the film-substrate interface. Second cross-section area “B” (Fig. 9b) shows a through-interface crack deviated 25° from the vertical. With the increasing normal load the cracking pattern changes and become more intensive. This is apparent from the cross-section area “C” closely before the fatal damage of the film (Fig. 9c), where several brittle through-thickness cracks are observed in the film. Extensive interference between median, Hertzian (conical) and lateral cracks in the substrate can also be recognized^123,124^.

**Figure 10:**
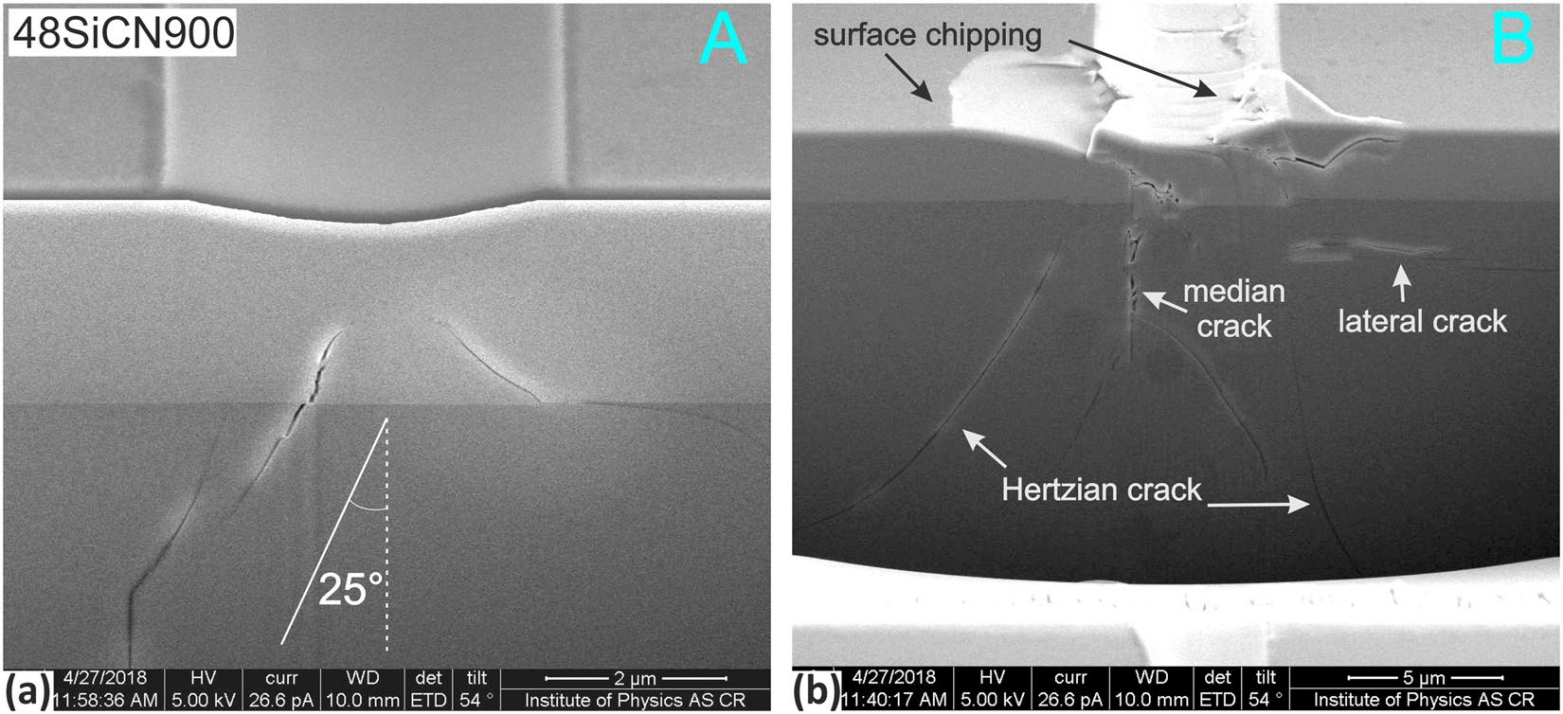
FIB cross sections in the 48SiC900 sample corresponding to the (**a**) the initial *L*_C1_ cracking and (**b**) distorted surface at the residual scratch end.

In case of the sample 48SiCN900 the first AE was detected in much latter part of the ramped scratch test, i.e. at higher normal load. Although no traces of cracking were visible on the film’s surface, the cross section “A” (Fig. 10a) shows two through-interface cracks that do not reach the film’s surface. Only faint cracking within the residual groove and film cleavage at its edge was observed at the very end of the residual scratch. However, the corresponding cross-section “B” (Fig. 10b) again revealed a combination of different types of pronounced cracks. In contrast to SiC film, no through-thickness cracks were observed.

The FIB milling clearly point out on different response of the SiC and SiCN films response to scratch loading. In case of the 00SiC900 sample the film-confined cracking taking place at lower load was initially observed, while through-interface cracking was identified as the first detectable type of failure for the 48SiCN900 sample. This points out on the higher fracture resistance of the SiCN films where higher strains are necessary for crack formation.

#### Multi-pass wear test

**Figure 11:**
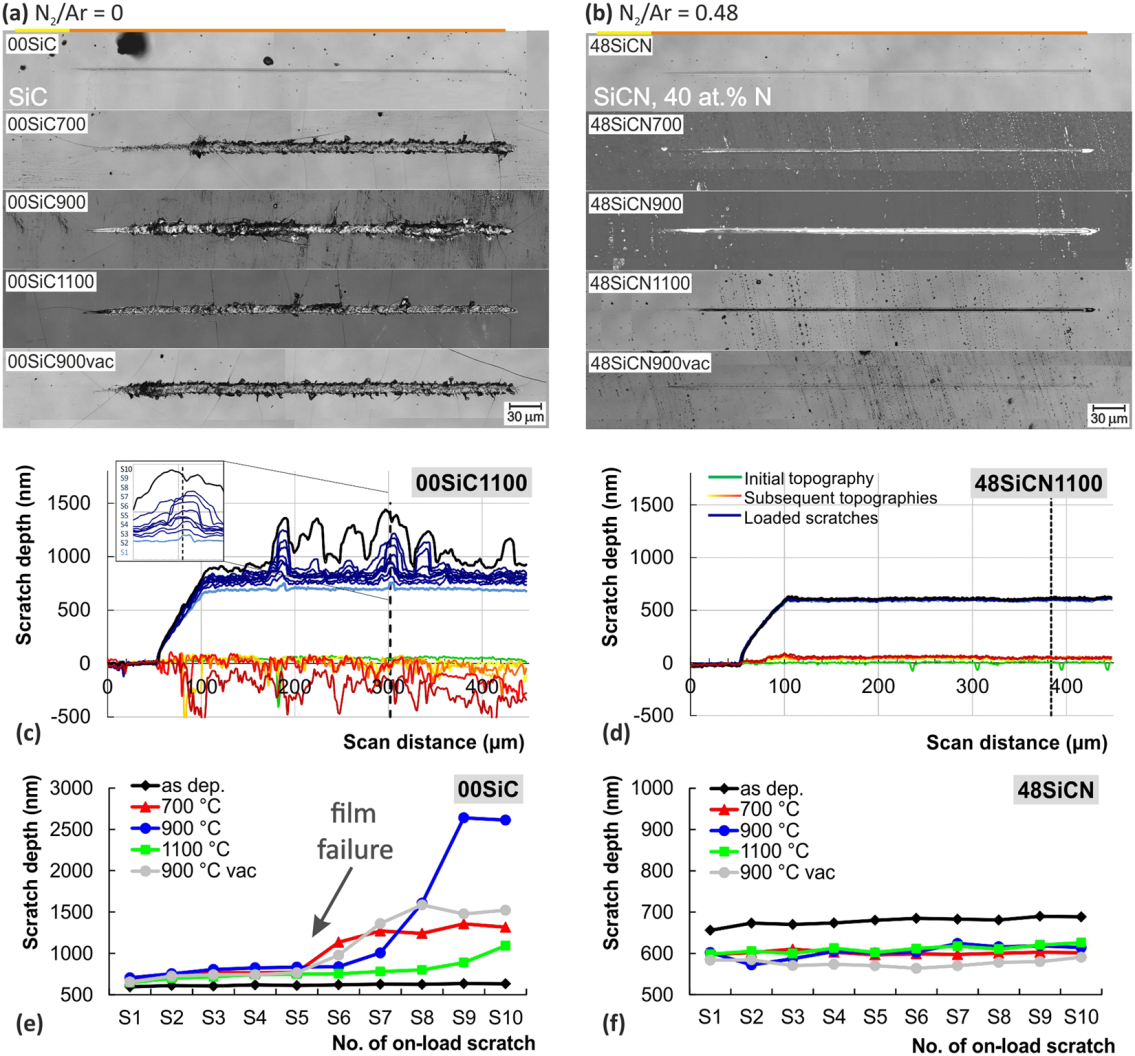
Comparison of multi-pass wear test results for 00SiC sample without nitrogen and 48SiCN sample with maximum nitrogen content, both before and after complete set of annealing. The results are in the form of microscopic images of residual scratches (**a**,**b**), representative depth records for 00SiC1100 and 48SiCN1100 samples (**c**,**d**), and depth increments of on-load curves (**e**,**f**).

The multi-pass wear tests were used to mimic the very often in-service scenario of repetitive sub-critical stresses from single asperity contact^102^. They were performed as a sequence of alternating multiple scratch and topography passes, where the subcritical constant load of 300 mN that was chosen based on evaluation of ramped scratch test. The illustrative microscopic images of residual grooves for the as deposited and annealed SiC films are shown in Fig. 11a. The residual wear track of the as deposited SiC film shows smooth surface with only faint plastic deformation, while apparent wear can be observed for all the annealed films. Air annealing at 700 °C led to the increased brittleness manifested by the occurrence of tensile cracks and cracks perpendicular to the scratch direction spreading out of the wear track. Increase of annealing temperature to 900 °C led to more pronounced chipping at the edges of residual groove. On the other hand, the SiC film annealed at 1100 °C exhibits wear track with more compact edges. The wear track for the vacuum annealed 00SiC900vac film resemble to the one for the 700 °C annealed film. This similarity may be related to the limited effect of oxidation top layer for the 700 °C air annealed film. It should be noted that this behavior clearly correlates with the critical load values (Fig. 5).

Small incorporation of nitrogen into the films structure led to the great increase of wear resistance of the annealed films. Already the 04SiCN sample with 14 at.% of nitrogen did not exhibited any film failure or cracking during the wear test. Only gradual plastic deformation was observed. In fact all the other samples with higher nitrogen content (08SiCN to 48SiCN) showed similar results. Figure 11b shows residual scratches after wear tests for the 48SiCN film with the highest N content before and after annealing. No film damage can be observed regardless of the annealing conditions.

Figure 10c,d show representative records of on-load and topography depths for 00SiC1100 and 48SiCN1100 films after annealing at 1100 °C in air. The 00SiC1100 sample exhibits distinctively developed topography as well as on-load depth profiles reflecting the gradual damage of the film during the test. On the contrary, all the depth records for the 48SICN1100 film are smooth. It should be noted that all the films containing nitrogen exhibit similar character of depth records. Hence Fig. 11 provides a useful comparison of the N effect on wear resistance for annealed SiC and SiCN films.

In order to quantify the repetitive scratch tests the depth-load records were evaluated based on the depth increments at specific distances from the beginning of the test. Based on the analysis of these increments, it is possible to explore the films’ gradual surface degradation and hence their durability. The on-load depth increments reflect both the elastic and plastic deformation of the material, while it is only the plastic deformation in case of topography depths increments. Besides, the initial topography scan performed at the beginning of the wear test, can be used for surface roughness analysis.

The evolutions of representative on-load depth increments for SiC and SiCN films before and after annealing clearly point out on the different performance of tested films, see Fig. 11e,f. The on-load depth increment profile for the as deposited SiC (green line in Fig. 11e) shows a gradually increasing trend that is symptomatic for plastic deformation. More developed profiles with sudden depth drops representing the film damage can be seen for the annealed films. The film annealed at 700 °C in air and at 900 °C in vacuum failed during the sixth on-load scratch (S6), the film annealed at 900 °C in air during the seventh on-load scratch. Rather gradual depth increase is observed for the SiC film annealing in air at 1100 °C. This is consistent with the microscopic observation and can be attributed to different stress redistribution and accommodation mechanics during scratches coming from the more developed short range ordering in film structure after higher temperature annealing and beneficial role of SiO_x_ tribolayer, especially for films annealed at higher temperatures in air.

The depth increment curve for the as deposited 48SiCN film shows only small depth increase, see Fig. 11f. The higher penetration depths, in comparison to pure SiC, stem from the lower hardness of the SiCN films, see Fig. 2. Air annealing led to the films structural changes and development of SiO_x_ top layer that in turn improved the wear resistance. This can be clearly seen from the shifting of on-load depth increments profiles to lower depths regardless of annealing temperature. The lower depth profile for the vacuum annealed films may be linked to the absence of softer SiO_x_ layer.

## Conclusions

The tribo-mechanical properties of SiC_x_N_y_ (y ≥ 0) thin films before and after air (700, 900 and 1100 °C) and vacuum (900 °C) annealing were explored in terms of N content and annealing temperature. The DC reactive magnetron sputtered films (2.2–2.7 µm) were deposited at RT (without any intentional heating) on Si substrates. The effect of deposition conditions, composition and structure on scratch resistance, fracture susceptibility and wear were discussed.

The composition of the amorphous as deposited films changes from pure SiC to SiCN films with the N content up to 40 at.%, while the Si/C ratio remains close to one. The compressive internal stress in the films changes from 1.7 to 1.1 GPa with the N content. The microstructure of the a-SiC films is composed of the Si-rich and C-rich regions and the a-SiC clusters. Introduction of nitrogen promotes formation of Si-N, C-N and Si-C-N bonds. Triple C≡N bonds are also form in the films with N contend above 30 at.%. All the SiCN films preserved amorphous structure even after annealing at 1100 °C. On the other hand, the traces of partial crystallization in SiC clusters were observed for the SiC film after annealing at 1100 °C.

In ramped load scratch tests both the critical loads representing the first failure in the film-substrate system and the total damage of the film increase with the N content in the films. The positive effect of nitrogen incorporation into the films structure is especially strong after thermal annealing. This improvement in scratch and fracture resistance stems from the hindering of the crystallization of SiC clusters due to the presence of N atoms and formation of multiple C≡N bond that is termination one for carbon. Different stress redistribution and accommodation mechanics then take place. The most fracture resistant films Si_0.32_C_0.32_N_0.36_ and Si_0.30_C_0.30_N_0.40_ exhibit N/Si > 1. Besides, the formed passivation SiO_x_ top-layer, that serves as a tribolayer, plays an important role in the improvement of the scratch resistance of the SiCN thin films. Its thickness grows for samples with higher nitrogen content. Vacuum annealed films exhibit much higher susceptibility to crack propagation despite their slightly higher hardness in contrast to air annealed films. The wear resistance of the SiC and SiCN films, evaluated via a repetitive scratch test, is consistent with the ramped scratch test findings and increases with the growth of N content and annealing temperature.

Comparison of the nanoindentation results and the scratch test performance clearly point out on the best tribo-mechanical performance of the a-Si_0.32_C_0.32_N_0.36_ films deposited at N_2_/Ar = 0.32 with sufficiently high hardness of approx. 20 GPa and sufficient level of ductility. Films with lower nitrogen content exhibit higher hardness on one hand, but higher brittleness on the other hand.

The dynamics of the film-substrate system failure was explored using the complex approach based on combination of visual observation of the residual groove, depth change record and acoustic emission record combined with FIB cross-sectioning of the residual scratch groove. This proved to be vital for reliable evaluation of the micro-scratch and micro-wear test since neither visual observation nor depth change record can solely render the comprehensive view of the process. AE records provide unique information about the subsurface failure in the film-substrate system, which are unattainable by traditional methods. The advanced analysis of the AE signal in time and frequency domain allowed distinguishing between the film and substrate damage and elucidated the processes going on during the scratch test.

The FIB milling revealed different initial cracking pattern for SiC and SiCN induced by scratch loading. Film-confined cracking was observed for SiC film at lower load, while through-interface cracking was identified for the SiCN film at higher load. This points out on the higher fracture resistance of the SiCN films where higher strains are necessary for crack formation.
